# Bioethics curriculum in medical schools in Portuguese-speaking countries

**DOI:** 10.1186/s12909-022-03250-9

**Published:** 2022-03-22

**Authors:** Ana Carolina Alvares Lavigne de Lemos Tavares, Ana Gabriela Alvares Travassos, Francisca Rego, Rui Nunes

**Affiliations:** 1 Faculty of Medicine of the University of Porto, Porto, Portugal; 2 Department of Life Sciences of the State University of Bahia, Bahia Salvador, Brazil

**Keywords:** Curriculum, Bioethics, Medical education, Cultural diversity

## Abstract

**Background:**

A curriculum is a fundamental tool for educators, and teaching bioethics is fundamental to good medical practice. Studies report a lack of consensus on the teaching of bioethics in undergraduate medicine, and a critical issue is that there remain no minimum curricular parameters. This study performed an analysis between the bioethics curricula of the medical schools of Brazil and Portugal and UNESCO’s Core Curriculum, in addition to proposing key criteria for designing a core bioethics curriculum.

**Methods:**

This is a cross-sectional, descriptive study that analyzes the bioethics curricula of the medical schools in Brazil and in Portugal. The design of the study includes a qualitative summative content analysis-based approach and a quantitative analysis by means of descriptive statistics.

**Results:**

Bioethics is taught in both Brazil and Portugal in a diversified way. The results showed that 65.5% of the medical schools analyzed provided at least the 30-h minimum workload recommended by the UNESCO Core Curriculum. Furthermore, bioethics sporadically offered at the end of the medical program in the vast majority of schools studied.

**Conclusions:**

The most important points to take away from this study are the diversity of the curricular structure of bioethics courses and the lack of formalization of bioethics in the curricula of medical schools in Brazil and Portugal. Given the value of bioethics in clinical practice, we propose that medical schools in Portugal and Brazil update their curricula to encompass minimum criteria, which should be similar to one another and based on common sources, but which should also be tailored to each culture.

## Background

A curriculum is a fundamental tool that educators can use to communicate what, how, and at what point in the M.D. program the essential content of each discipline should be taught [1]. This also applies to courses on bioethics, which is the study of universal bioethical principles and their practical application [2] and in recent years, profound discussions about how best to teach bioethics to future physicians have become increasingly common.

Teaching bioethics is fundamental to good medical practice, as it combines the application of scientific knowledge with a respect for the values and preferences held by the patient, attempting to render them active participants in the care process. Technical issues aside, the teaching of bioethics must overcome a common problem, namely, the considerable influence of the hidden training curriculum, given that the quality of this influence cannot be controlled [3]. Some studies refer to the need for additional investments in the formal teaching of ethics in medical courses [4, 5] to overcome important problems—such as the aforementioned hidden curriculum—which may undermine the consistency of bioethics teaching across medical programs [6].

The basis for a theory on curricula would be the definition of what knowledge should be taught and what skills would be acquired as a result of a theoretical selection of content that possesses the greatest pedagogical-didactic value to the learning-teaching process [7]. Studies reinforce the comprehensive and structured nature of the curriculum based on a clear and strategic vision of the holistic and inclusive development of all students, aiming to provide them with the essential knowledge, skills, and competencies and with a high-quality education [8, 9]. According to Young [10], since curricula have been in use, its critical and normative goals have been separated, requiring the teachers to understand curricula as specialized forms of knowledge in order to be able to develop better courses and broaden learning opportunities. Thus, a broader conception of curricula, put forth by Aguiar [7] in Macedo (2002), provides an important reflection in which the curriculum serves as a foundation upon which to construct a broader educational theory wherein several subjects interact in educational spaces. In addition to having a solid foundation, a curriculum must also be flexible enough to be effectively implemented within the educational system [11]. An American study, published in 2015, reports a lack of consensus regarding goals/objectives, teaching methods and assessment strategies on teaching of bioethics in undergraduate medicine and suggested that the bioethics curriculum can be improved by focusing it on professional training [12]. Another study carried out in the United Kingdom in 2016 [13] demonstrates the shift in several countries towards seeking to unify the bioethics curriculum. One important aspect to consider is that the hidden curriculum in bioethics education—both in terms of pedagogy and content point of view—must be made explicit in order to help students recognize and understand the cultural context, so that the most favorable methods can be determined to achieve the desired educational outcomes [14]. According to Changiz et al. [15], the monitoring and management of undergraduate medical education curricula are essential factors for that medical training to be efficient.

A crucial aspect raised by our team and others is what knowledge the curriculum should comprise [7, 10]. Young [10] suggests that those responsible for designing curricula participate in a process of recontextualization, understood to represent how elements of subject knowledge are incorporated into the curriculum for a heterogeneous audience; it is imperative that these elements emphasize the purpose of the curriculum so that it truly promotes the necessary conceptual progression and epistemic growth. It is important to note that each society lays the groundwork for the type of social imaginary it desires for its current and future generations by relying on the knowledge and competencies gained throughout the curriculum [11].

Given these considerations, formal education in bioethics to future physicians is urgently needed in today’s society, and one of the largest barriers to this end is the lack of minimum curricular parameters—although it is understood that bioethics issues should be similar across the current liberal, democratic context.

Therefore, this work aims to perform a comparative analysis of the bioethics curricula of medical schools in Brazil and in Portugal and UNESCO’s proposed Core Curriculum in Bioethics Education. It also aims to propose key criteria for designing a nuclear bioethics curriculum for Portuguese-speaking countries. The UNESCO Bioethics Core Curriculum was selected as a comparison index because it is a highly comprehensive curriculum grounded in the UNESCO Universal Declaration of Bioethics and Human Rights [2], which means that its roots are grounded in the universal humanitarian principles of human dignity, human rights, nondiscrimination, and respect for the environment and the commonwealth of life. In short, it is a universal curriculum for a global community and is in accordance with the universalist culture of Portuguese-speaking countries.

In this study it is not made a conceptual distinction between bioethics, medical ethics, or even biomedical ethics. Although there might exist different perspectives on this issue, namely in the European and American traditions of bioethics, for the purpose of this study these concepts are used interchangeably because otherwise it would be difficult to analyze different international studies in this area. Also, it is assumed that bioethics in the medical setting obviously comprises medical ethics.

## Methods

This cross-sectional, descriptive study analyzed the bioethics curricula of the medical schools in the Portuguese-speaking countries of Brazil and Portugal. Indeed, these two countries share not only a language, but also common cultural traditions and similarities in medical training. Nevertheless, these two countries have important geographical differences; namely, Brazil’s large land area requires a more heterogeneous medical teaching and training curriculum. The fact that both Brazil and Portugal are liberal democracies with highly similar constitutional laws—grounded in the respect for human dignity and fundamental rights—was a determinant in the development of a secular bioethics in both countries, which share a common approach to bioethics.

### Sample

The curricula sample was two medical schools from each federal state in Brazil (*n* = 52) and all the medical schools in Portugal (*n* = 07), for a total of 59 curricula.

Regarding Brazilian medical schools, two medical schools from each federal state were selected for the sample, due to the continental dimensions of the country and its great regional heterogeneity, namely physical, economic and cultural. In 2020, Brazil had 357 medical schools [16]. The criterion used in this study to establish which medical schools would be considered for inclusion in our study in Brazil was the top two per federal state in ENADE (National Student Performance Survey) ranking [17], a periodic and compulsory evaluation carried out by the Ministry of Education (MEC) of the country in which performance is expressed through concepts and ordered on a Likert scale (1 to 5). Given that our study is the first of its kind in this country, focus was placed on the medical schools that are of similar quality due to data accessibility. Moreover, the selection of top Brazilian medical schools has an added value not only to medical teaching in this country—as lower ranked medical schools try to improve their rank via the federally regulated accreditation process—but also to low- and middle-income Portuguese speaking countries that frequently consider top ranked Brazilian (and Portuguese) universities as the standard in benchmark comparisons.

Data collection began in July 2018 until April 2019 and was initiated with a direct search of university websites. In schools that did not publish teaching objectives and/or educational projects for the courses available on their respective websites, e-mails were sent to the medical course coordinators requesting the course syllabus and/or equivalent document plans explaining the content taught, course hours of contact per student, and distribution throughout the course of the subjects of bioethics, medical deontology, professional ethics, or equivalent. Of these, only one college in the North Region of Brazil did not respond to the e-mail and was excluded from the study. Ultimately, the final sample comprised 51 curricula from Brazil and 7 from Portugal, totaling 58 curricula.

### Data analysis

The study design included a summative qualitative content analysis-based approach. A summative approach to qualitative content analysis quantifies words or manifest content, as well as latent meanings, to explore the contextual use of those words/content [18, 19]. In this study, this process consisted on identifying and counting specific words or content present in the curricula selected, using the UNESCO proposed Bioethics Core Curriculum [20] as predetermined checklist. For descriptive statistics and absolute and relative frequencies, data were organized and analyzed in Microsoft Excel 2016.

The Bioethics Core Curriculum (UNESCO, 2008) [20] aims to introduce the bioethical principles of the Universal Declaration on Bioethics and Human Rights [2] to university students with content based on the principles adopted by UNESCO. An important characteristic of this curriculum proposal is that it does not impose a particular model or a specific view of bioethics; rather, it articulates ethical principles that are shared by scientific experts, policymakers, and health professionals from various countries differing widely in cultural, historical, and religious backgrounds [20]. Furthermore, it emphasizes the equal dignity of all human beings and the respect for the fundamental rights of each person.

The main variables analyzed were: structure of the Medical Degree (M.D.) in each country (years of the course, pre-clinical vs. clinical cycle and duration of bioethics teaching in years along the medical degree); bioethics teaching hours (hourly load in the curricula); point in the program at which bioethics is offered (preclinical vs. clinical cycle; per year of the medical course); and the bioethics teaching content included in the curricula. According to the guidelines of the Core Curriculum, the minimum hourly load is indicated as 30 total hours of contact [20]. Regarding the point in the program at which the subject is offered, an ideal moment is not described, although the authors mention that while it can be introduced in the pre-clinical phase of medical education, it should be implemented toward the end of the clinical phase, because learning about bioethics at this timepoint appears to be more effective [20]. With respect to course content, we found that it was based on the principles endorsed by UNESCO [20] and organized into 17 domains (Table 1).

**Table 1:** Core curriculum content

N°	Domains
1	What is ethics?
2	What is bioethics?
3	Human dignity and human rights
4	Benefit and harm
5	Autonomy and individual responsibility
6	Consent
7	Persons without the capacity to consent
8	Respect for human vulnerability and personal integrity
9	Privacy and confidentiality
10	Equality, justice and equity
11	Non-discrimination and non-stigmatization
12	Respect for cultural diversity and pluralism
13	Solidarity and cooperation
14	Social responsibility and health
15	Sharing of benefits
16	Protecting future generations
17	Protection of the environment, the biosphere and biodiversity

We compared these domains with the contents offered by the medical schools to verify whether each related topic was addressed by the curriculum.

The inclusion criterion for the contents was the presence of terms similar to the description of the 17 content themes proposed in the UNESCO Bioethics curriculum [20]. Given that not all curricula used the same terms as the domains proposed in the Bioethics Core Curriculum [20], the syllabi were also reviewed for alternative terms that corresponded to the pre-established domains. The content found was then quantified in terms of frequencies for each domain. Data were extracted by two researchers independently, it was discussed together and the final decision was then registered under each domain. In case of different opinions, it would be solved by reaching a consensus with the collaboration of two coordinators. It is noteworthy that the curricula were analyzed not only to identify bioethics disciplines, but also bioethics contents taught throughout the several disciplines and/or modules of medical graduation.

## Results

### Structure of the medical degree

To obtain an M.D. degree in Brazil or Portugal, a 6-year program is required comprising both theoretical and practical classes. During this period, the program is divided into a pre-clinical and clinical cycle, which differ in the 2 countries; the clinical cycle in Brazil begins in the 3rd year, while in Portugal it begins in the 4th year of the course. Another difference is that the last year of the course in Portugal is reserved for the supervised internship, which in Brazil corresponds to the internship that takes place in the last 2 years of the course.

Concerning the duration of bioethics teaching, our results indicated that in 50% (*n* = 29) of the medical schools analyzed, bioethics was taught predominantly during a single year of the M.D. program; more specifically, this was the case in 52.9% (*n* = 27) of medical schools in Brazil and 28.6% of (*n* = 2) medical schools in Portugal. Furthermore, 24.1% (*n* = 14) of the schools analyzed offered bioethics content in the curriculum during 2 years of the M.D. program: 23.5% (*n* = 12) of schools in Brazil and 28.6% (*n* = 2) of schools in Portugal. Additionally, 7.8% (*n* = 4) of medical schools in Brazil and 14.3% (*n* = 1) in Portugal taught bioethics content throughout 3 years of the curriculum, and 9.8% (*n* = 5) of the Brazil medical schools taught bioethics throughout 4 years of the curriculum. A total of 3.4% (*n* = 2) medical schools had bioethics content present throughout 5 years of the medical curriculum: 2% (*n* = 1) of the schools in Brazil and 14.3% (*n* = 1) of the schools in Portugal. Lastly, 14.3% (*n* = 1) of the medical school in Portugal offered bioethics content throughout the 6 years of M.D. degree.

### Bioethics teaching hours

Among the Brazilian medical schools analyzed, curricula were observed to be predominantly organized into thematic modules or axes in 62.75% (*n* = 32) of the faculties, without specific subjects necessarily included in the curriculum structure. Of these, some schools described the hourly load destined for each topic, while others only mention the presence of the topic. This aspect of the organization of the curricula rendered analysis of the weight that the topic of bioethics carries difficult, because the specific hourly load assigned to it is diluted among the various topics of the modules. As such, after analyzing the presence of the topic of bioethics in the curricula, a workload of over 30 h (the minimum recommended by the Core Curriculum) was identified in 60.8% (*n* = 31) of the analyzed curricula (Table 2).

**Table 2:** Distribution of curriculum hours by country, in absolute and relative frequencies, n(%)

Countries	No workload	Hourly workload < 30 h	Hourly workload of ≥30 h
Brazil	2 (4.0)	18 (35.2)	31 (60.8)
Portugal	0	0 (0)	7 (100)
Total (*N* = 58)	2 (3.5)	18 (31.0)	38 (65.5)

Of the seven Portuguese medical schools analyzed, five organized their curricula by disciplines. All Portuguese medical schools offered the subject of bioethics with a minimum hourly load of 90 h in 1 year, with some offering up to 180 h.

Our analysis revealed that all universities in Portugal analyzed explicitly state the minimum hourly load recommended by UNESCO’s Core Curriculum in their formal curriculum documents,60,8% (*n* = 31) of the universities in Brazil analyzed clearly show that this subject is addressed in their formal curricula, with the appropriate hourly load in accordance with this recommendation.

### Point in the program at which bioethics is offered

The distribution of the bioethics content offered in the curriculum in either the pre-clinical and clinical cycle differs between the two countries: it was more common for the medical schools to offer bioethics content only in the pre-clinical cycle in both Brazil (37.3%; *n* = 19) and Portugal (71.4%; *n* = 71.4%). The number of medical schools that offered bioethics content only in the clinical cycle was smaller: 29.4% (*n* = 15) for schools in Brazil and 14.3% (*n* = 1) for schools in Portugal. Nevertheless, some medical schools offered bioethics content in both the pre-clinical and the clinical cycle: 29.4% (*n* = 15) for schools in Brazil and 14.3% (*n* = 1) for the schools in Portugal. Furthermore, 3.9% (*n* = 2) of medical schools in Brazil did not offer bioethics’ content in either cycle.

The point in the program at which bioethics is offered varies greatly among the schools analyzed. Table 3 displays the distribution of bioethics curricular units per year in the medical schools of each country studied.

**Table 3:** Distribution of bioethics content across the medical schools of each country, n(%)

Countries	No. of Univs.	1st Year	2nd Year	3rd Year	4th Year	5th Year	6th Year
Brazil	51	27 (52.9)	17 (33.3)	14 (27.5)	23 (45.1)	3 (5.9)	4 (7.8)
Portugal	7	3 (42.9)	3 (42.9)	5 (71.4)	4 (57.1)	4 (57.1)	1 (14.3)
Total	58	30 (51.7)	20 (34.5)	19 (32.8)	27 (46.6)	7 (12.1)	5 (8.7)

In the Brazilian medical schools, bioethics is most offered is in the 1st year (52.9%; *n* = 27) and 2nd year (33.3%; *n* = 17); both periods during which students are still quite immature. We also found that bioethics rarely appears as a standalone topic in the formal curriculum of the medical internship years, during which students experience privileged moments of contact with patients. Only three schools describe this theme in the 5th year, which represents 5.9% (*n* = 3), and four schools in the 6th year, representing 7.8% (*n* = 4) of schools.

In Portugal, the point in the program at which the subject of bioethics is most often taught is in the 3rd year, corresponding to the final year of the pre-clinical cycle (71.4%; *n* = 5). Of the seven medical schools in the country, four begin offering the subject starting in the 3rd year, a period that marks the end of the pre-clinical cycle and precedes what are considered “clinical” years, in which students begin having direct contact with patients. It is worth highlighting the fact that Portugal has a medical school that formally includes the subject in the curriculum for every year of the medical course.

### Bioethics teaching content in the curricula

We collected data by comparing the explicit contents in the curricula of the medical schools of Portugal and Brazil, with the 17 content themes organized based on the curriculum proposed by UNESCO.

An important fact to highlight is that all Portuguese schools (100%; *n* = 7) offered the subject “What is ethics?”, while only 66.7% (*n* = 34) of Brazilian schools did.

The second most frequently offered topic in the curricula of Portuguese schools are those of “autonomy and individual responsibility” and “respect for human vulnerability and personal integrity”, which were explicitly described in 85.7% (*n* = 6) of school curricula. “What is bioethics?” is the third most frequently offered topic, found in 71.4% (*n* = 5) of schools, along with “human dignity and human rights”, “consent”, and “people who are unable to give consent”.

**Fig. 1:**
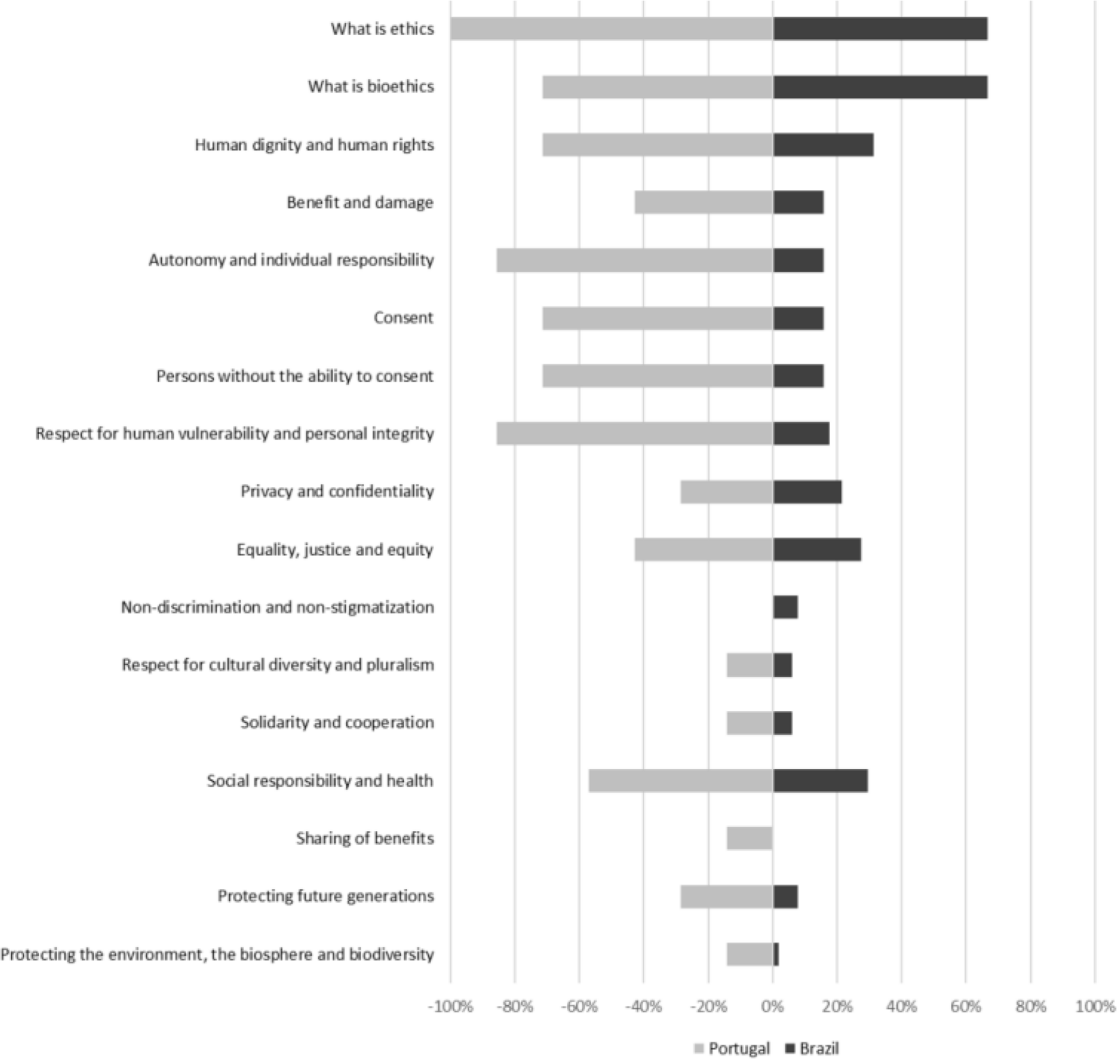
Distribution of curricular contents proposed by UNESCO in medical schools in Brazil and Portugal (%)

In Brazil, “What is ethics?” and “What is bioethics?” are the most prevalent topics, appearing with similar frequency in the curricula of 66.7% (*n* = 34) of the schools analyzed. The second most prevalent topics are “human dignity and human rights” with 31.4% (*n* = 16), “social responsibility and health” with 29.4% (*n* = 15), and “equality, justice and equity” with 27.5% (*n* = 14) Figure 1.

## Discussion

The teaching of bioethics in Brazil and in Portugal is characterized by heterogeneity, and the definition of minimum curricular parameters is an important challenge for medical educators to tackle. One of the pillars in the transformation of the curricula discussed by the IBE (International Bureau of Education, 2017) [11] is precisely the challenge of determining what knowledge is relevant and should be included in a given curriculum, providing consistent guidance while simultaneously ensuring that it possesses the flexibility necessary for a modern curriculum to keep pace with the rate of human knowledge growth in today’s society.

Our research has shown that the content offered in the analyzed curricula of some medical schools in Brazil that were part of this study is not made explicit. Nunes (2014) demonstrates the need to seek a common platform for the resolution of bioethical conflicts, especially in the face of the cultural pluralism that we are experiencing today. The author further reinforces that the doctor–patient relationship, as well as modern society, has undergone several transformations and have failed in the process at adopting a uniform ethical framework—one in which physicians and patients are active agents in decisions involving ethical values and dilemmas [21]. In the 1990s, a consensus statement was drafted in the United Kingdom in which medical schools agreed on the topics that should comprise the core of a basic curriculum of bioethics and law. In addition to the minimum content proposed, the group recommended that bioethics and law should be included throughout the entire clinical cycle [22].

A study carried out in 2016 in Canada [14] posits that it is important to understand the relevant and common issues of a culturally diverse student body in order to define the specific high-impact topics to be addressed in bioethics education. This aspect is directly related to the similarities and differences found in the topics that were most frequently offered at the Brazilian and Portuguese medical schools analyzed. The most addressed conceptual topic in both countries is “What is ethics?”; however, the most discussed topics in the medical schools in Brazil are those that deal with collectivity, such as “human dignity and human rights,” “social responsibility and health”, and “equality, justice, and equity.” In Portugal, however, students are more exposed to issues related with individual responsibility in the context of medical practice, such as “autonomy and individual responsibility” and “respect for human vulnerability and personal integrity”. This may be explained by the greater maturity of bioethics education in Portuguese schools, following the philosophical tradition underpinning the approach to bioethics in continental Europe, which emphasizes and questions human actions and the principles that determine the morality of action [23]. This is different from Brazilian medical curricula, in which the inclusion of bioethics is slightly more recent and seeks to guide health training towards understanding the health priorities of the most vulnerable communities by informing education policy with health policy in implementing the Unified Health System [24, 25]. There is also a shortage of data on which areas of undergraduate training bioethics is lacking [26]; however, there has been a growing trend for some time toward allowing bioethics education to permeate students’ daily experiences, so that it affects their clinical interactions and personal experiences as doctors [27]. Some studies have reported that incorporating bioethics training into clinical training increases student confidence and improves decision-making [28, 29].

The present research revealed that most Brazilian and Portuguese medical schools studied offer bioethics teaching in their curricula for 1–2 years, but the occurrence of this teaching differs greatly relative to the period in the M.D. degree in which this theme is offered. Furthermore, the teaching of bioethics is mostly offered exclusively during the pre-clinical cycle in both Brazil and Portugal; nevertheless, several Brazilian medical schools were found to offer bioethics in both pre-clinical and clinical cycles. Although UNESCO’s Core Curriculum does not formally recommend the best timepoint in the M.D. program at which to offer bioethics as a subject [20], it is sporadically offered in the final years of the medical program in most of the schools analyzed, which is exactly the stage at which students will have greater opportunities to experience ethical conflicts in managing the cases of prospective patients. A 2016 study evaluating UK bioethics curricula found that the Institute of Medical Ethics (IME) recommendations were not followed in all cases and that ethics teaching was not properly integrated into clinical practice, sparking the authors to contend that patients may be treated in the future by physicians inadequately prepared to make ethical decisions in clinical practice [30].

A concerning finding of our research was the irregularity with which the subject of bioethics was formally offered at the final stage of the medical degree, especially given that American studies had already demonstrated the importance of horizontally and vertically integrating the teaching of ethics into the curricula of medical schools. These American schools offered it every year during the degree program, especially during the clinical years, during which students begin to develop their personal style of practice [27, 31]. Studies reinforce the need for medical curricula to extend beyond a conceptual and analytical approach to bioethics, to provide students with practical approaches to deal with everyday ethical conflicts more effectively [26, 32]. A study in Brazil reported that bioethics was taught during the medical internship period in fewer than 30% of schools [33], whereas another reported that standalone bioethics subjects are offered at the start of the program [34]. Although some schools provide adequate conceptual training, students report feeling unprepared to deal with practical situations involving ethical conflicts [32, 35, 36]. Attempts to implement an integrated bioethics curriculum have shown that the integrated approach provides skills to effectively contend with moral dilemmas that commonly arise in clinical practice [31]. As in our study, the aforementioned studies reinforce the need to pay attention to the fact that the Brazilian curricula analyzed had little formalized training of bioethics-related subjects, which were instead diluted across other disciplines and/or modules. A study done in 2019 identified that the lack of standardized curricula to be followed is one of the main barriers to implementing bioethics education [37].

In addition to the analysis of the content and of the timepoint in the degree program at which the subject of bioethics is offered, the results of this research showed that 65.5% (*n* = 38) of medical schools in Portugal and Brazil offered the minimum 30-h workload recommended by the UNESCO Core Curriculum. A study conducted in Spanish medical schools found compliance with the 30-h minimum requirement of the UNESCO Core Curriculum throughout the country, in addition to highlighting the importance of all disciplines to be taught with bioethics in mind [38]. Interestingly, newer Spanish schools dedicated nearly double the hourly load to teaching ethical values than that offered in older schools. Another 2018 study analyzed 276 medical schools in Latin America and found that the hourly load for bioethics was strikingly low relative to the total degree program hours and suggested more cross-cutting teaching models throughout the program, even dedicating internship hours to the subject to facilitate clinical discussions based on real cases [33].

It follows that a set of criteria can be suggested for medical teaching in Portugal and Brazil, with possible future expansion to other Portuguese speaking countries in Africa and Asia. Namely:

- The existence of a standardized curriculum with sufficient flexibility to adapt to the specificity of each cultural background.
- The implementation of an updated curriculum in accordance with the current bioethical issues relevant in modern societies, namely the key criteria adopted by the UNESCO Core Curriculum.
- A minimum 30-h load.
- A longitudinal program offered every year of the medical course. Or at least formal training in the pre-clinical and in the clinical cycles.
- As a compulsory (not optional) subject.
- The bioethical curriculum should be developed and implemented in accordance with modern teaching modalities that promote the acquisition of knowledge and skills in bioethics.

Important limitations of this study include the researchers’ use of secondary data, which took the form of the curricular documents obtained from the schools recruited; the different forms of recording the information contained in these teaching plans; and, in the sample defined for the medical schools in Brazil, the inclusion of only the top-ranked schools from the federal states. The cultural differences between the countries involved in the analysis render data interpretation even more challenging. We also know that the minimum curriculum proposal needs to consider the social and cultural discrepancies between Brazil and Portugal and the adaptation of topics according to these discrepancies.

Due to the collective record of data, it was not possible to determine the level of agreement between the investigators’ interpretations. Future studies should calculate an agreement coefficient to control possible bias.

It is suggested that further studies explore whether there are other bioethics contents present in the curricula of medical schools besides the domains recommended by the UNESCO Bioethics Core Curriculum.

## Conclusions

The objective of this research was to compare the bioethics curricula of medical programs in Brazil and Portugal with UNESCO’s proposed Core Curriculum, as well as to propose key criteria that should comprise this curriculum. The most important variables found in our study refer to the diversity of the curricular organization regarding the subject of bioethics and the lack of formalization of the discipline in the aforementioned curricula. The curricula are expected to be updated in accordance with the current ethical issues affecting modern society and each culture; therefore, researchers have recommended that bioethical topics should remain similar across the current secular and democratic contexts of modern societies.

Given the valuable resource that bioethics represents for clinical practice, we recommend that future studies include all medical schools in Portuguese-speaking countries and that curricula from these schools should be kept up to date to include the minimum criteria, which should be reasonably similar and based on common sources that consider the cultural specificities of each culture. Studies involving medical teaching in Brazil and Portugal are important to all Portuguese-speaking countries, due to the example of best practices that might be given to countries with common roots and shared ideals. And because the interconnectedness and interdependency of higher education systems in all countries that use Portuguese as the leading language of instruction facilitates the implementation in the near future of integrated programs of this nature that have the objective of divulging and promoting essential bioethical principles.

Among these criteria, we suggest adopting the UNESCO Core Curriculum recommendation of a minimum 30-h load and formalizing the subject of bioethics in the curricula, preferably by offering it every year during the degree program and, if possible, as a compulsory subject. The curricula should adopt similar minimum content that would encompass the most prevalent domains, highlighting those described in the UNESCO Core Curriculum, such as conceptual aspects of ethics and bioethics, autonomy and individual responsibility, respect for human vulnerability and personal integrity, human dignity and human rights, social responsibility, and health, equality, justice, and equity. Its applicability in the clinical practice of future physicians could reflect such a change, and Portuguese-speaking universities could feasibly hold common forums in which new propositions could be debated.

## Data Availability

The datasets used and/or analyzed during the current study are available from the corresponding author on reasonable request.

## Abbreviations

UNESCO: United Nations Educational, Scientific and Cultural Organization
ENADE: National Student Performance Survey
MEC: Ministry of Education
IBE: International Bureau of Education
